# Hospital Architecture and Construction

**Published:** 1911-01-07

**Authors:** 


### Hospital Architecture and Construction.

[Communications on this subject should be marked "Architecture" in the left-hand top corner of the envelope.]

We gladly accede to a general wish to add to the paper a department on Hospital Architecture and Construction. This department will be placed in the hands of a commission of three, including eminent hospital architects who are responsible for some of the most important and best hospital work which the world has so far produced. It will be our aim and object to make this department of practical use to all who are engaged in the management of hospitals, and we shall be prepared to answer questions and to give every possible assistance in the solution of special problems with which the authorities of hospitals and kindred institutions may have to deal from time to time. We rely on the co-operation of our readers to keep us informed as to any new works, reconstruction, or new hospital buildings and appliances which may from time to time be contemplated or sanctioned. The subject is a wide one, and to make this department of the utmost practical use it is desirable that hospital managers generally shall make the fullest use of it and test it, so that it may become capable of meeting all requirements. Thus it should prove of real assistance to all types of hospitals and medical institutions for the reception of the sick.

### The New "Works Department."

In the best managed large hospitals, it is becoming the practice to do much of the repairs and minor building operations. It follows that articles of a practical character dealing with every branch of construction must prove generally welcome. Our aim will be to make the treatment of the subjects dealt with in this department not only practical but interesting. Having this in view, we shall welcome photographs of existing hospital architecture, both internal and external, which has a special interest or beauty. If the elevation, gardens, and grounds of a particular hospital are unusually attractive we should be glad to receive a photograph of any or all of them. Again, we desire to obtain photographs and elevations of interiors which have a special interest owing to their relating to a bygone period of hospital development, like that of the sisters' rooms with the peepholes looking into the wards, or of memorial tablets quaintly worded and containing an indication of the arrangements of the staff or the like. Then there are new features of construction and planning which may be specially attractive as exhibiting an attempt to take a step in advance. As an example, we may mention the Cardiff Infirmary, where the sisters' rooms constitute a special feature from their attractiveness, size, and hygienic completeness.

Then, again, there are many hospital officers and managers taking a special interest in their hospital who have introduced a number of useful appliances of patterns which are not generally known, but often possess features of great usefulness. Every hospital now has at least one member on the staff who is fond of photography, so that means are at hand in every hospital which should enable the secretary or resident medical officer to obtain a photograph of any portion of the buildings likely to prove of general interest or value. These are only a few directions in which existing hospital buildings, and the appliances they contain, may be utilised in an interesting way.

No such section as is here contemplated will be complete unless it deals, as we intend this to deal, with the subject of competitions and contracts, whilst notes upon hospital machinery and fittings must always prove helpful to all hospital workers who desire to keep their particular hospital as efficient as possible.

### Problems of Future Construction.

There are many problems of hospital construction about which information is incomplete. Amongst these are flat roofs for garden purposes in towns; the ideal hospital building in regard to air currents; the cost of heating by different methods, and the most economical and efficient of them; laundry fittings; plumbing work; the place of the electrician in hospital construction; lifts; gas as a motive power and for lighting, and its effects on the atmosphere; bell installations versus telephones; the ever-changing and developing requirements of the operation theatre; sanitary appliances, and the aid of architectural details to administrative efficiency. Amongst country hospitals especially we hope to make a very interesting section which will embrace the hospital garden, including a consideration of the position of trees and their effect on the atmosphere; the flower garden, the kitchen garden, and the method by which the one can be made to pay for the other.

In the course of our inspection of the hospitals and kindred institutions throughout the world, especially in recent years, we have been impressed by the fact that there are many points in general administration intimately affected by the works embraced in the section we are now considering which are generally unknown, to the no small disadvantage of hospital workers. Above all things our aim will be to make this section of the paper of the utmost practical value to hospital managers generally. We are convinced that the developments which have taken place in very many departments of construction and works during the last decade or two are not as widely known or as keenly appreciated as they deserve to be. All such matters will receive careful treatment, and with the co-operation of our readers we have no doubt that we shall be able in process of time to aid the hospital workers amongst them to introduce many economies, some beauties, and possibly here and there increased comforts and hygienic completeness without material expenditure and often with the ultimate result that whilst efficiency will be increased expenditure may be curtailed.

